# HDAC Inhibitors and RECK Modulate Endoplasmic Reticulum Stress in Tumor Cells

**DOI:** 10.3390/ijms18020258

**Published:** 2017-01-26

**Authors:** Yun Chen, Ya-Hui Tsai, Sheng-Hong Tseng

**Affiliations:** 1 Department of Surgery, Far Eastern Memorial Hospital, 21, Sec. 2, Nan-Ya South Road, Banciao, Taipei 220, Taiwan; ychen@mail.femh.org.tw (Y.C.); yahuitsai@gmail.com (Y.-H.T.); 2 Department of Chemical Engineering and Materials Science, Yuan Ze University, 135 Yuan Tung Rd., Chung-Li, Taoyuan 320, Taiwan; 3 Department of Surgery, National Taiwan University Hospital and National Taiwan University College of Medicine, 7 Chung-Shan S. Rd., Taipei 100, Taiwan

**Keywords:** histone deacetylase inhibitors, reversion-inducing cysteine-rich protein with Kazal motifs, endoplasmic reticulum stress, cancers

## Abstract

In the tumor microenvironment hypoxia and nutrient deprived states can induce endoplasmic reticulum (ER) stress. If ER stress is not relieved, the tumor cells may become apoptotic. Therefore, targeting ER homeostasis is a potential strategy for cancer treatment. Various chemotherapeutic agents including histone deacetylase (HDAC) inhibitors can induce ER stress to cause cell death in cancers. Some HDAC inhibitors can prevent HDAC from binding to the specificity protein 1-binding site of the promoter of reversion-inducing cysteine-rich protein with Kazal motifs (*RECK*) and up-regulate RECK expression. Up-regulation of RECK expression by HDAC inhibitors has been observed in various cancer types. *RECK* is a tumor and metastasis suppressor gene and is critical for regulating tumor cell invasiveness and metastasis. RECK also modulates ER stress via binding to and sequestering glucose-regulated protein 78 protein, so that the transmembrane sensors, such as protein kinase RNA-like ER kinase are released to activate eukaryotic translational initiation factor 2α phosphorylation and enhance ER stress. Therefore, HDAC inhibitors may directly induce ER stress or indirectly induce this stress by up-regulating RECK in cancer cells.

## 1. Endoplasmic Reticulum Stress in Cancers

The endoplasmic reticulum (ER) is an important cellular compartment involved in protein synthesis and maturation. Protein maturation requires the coordinated activity of many chaperones and folding enzymes [1,2]. When the number of unfolded proteins exceeds the capacity of the ER, cellular protein homeostasis is disrupted and ER stress occurs, leading to the accumulation of unfolded or misfolded proteins [1,2,3]. To reduce the excessive protein load, cells activate the unfolded protein response (UPR), which causes transient attenuation of protein translation, degradation of misfolded proteins, and induction of molecular chaperones and folding enzymes to augment the ER capacity for protein folding and degradation [2]. The UPR is controlled by glucose-regulated protein 78 (GRP78) and three different ER transmembrane sensor proteins: protein kinase RNA-like endoplasmic reticulum kinase (PERK), inositol-requiring enzyme 1 (IRE1), and activating transcription factor-6 [1,3,4,5]. GRP78 acts on newly-synthesized proteins by chaperoning them through folding, assembly, and translocation across the ER membrane [6]. In normal and non-stressed cells, GRP78 binds to ER transmembrane sensor proteins and maintains them in an inactive form [1,4,5]. During ER stress, an increase in unfolded substrates leads to the sequestration of GRP78, releasing the sensors to initiate UPR signals [1]. If this ER stress is not relieved, the injured cells may become apoptotic [2]. ER stress can be induced by various insults, such as hypoxia, reactive oxygen species, nutrient deprivation, disruption of calcium homeostasis, inhibition of protein glycosylation or disulfide bond formation, and viral or bacterial infection [2,3].

ER stress also plays an important role in tumor cell survival, tumor progression, angiogenesis, metastasis, and drug resistance; common conditions in the tumor microenvironment such as hypoxia, reactive oxygen species, and nutrient deprivation can trigger the UPR [2,3,4,6,7]. Tumor cells often produce more mutant proteins than the normal ER capacity can handle because of rapid biosynthesis in cancers, and eventually the nutrient requirements exceed the capacity of the vascular supply, making the tumors hypoxic and causing tumor cell apoptosis [1,5,6]. During ER stress, GRP78 increases so that tumor cells can adapt to the chronic ER stress [2,4]. Increased GRP78 expression has been observed in various cancer cell lines and human cancer specimens, such as bladder, breast, lung, and stomach cancers, glioma, melanoma, and epidermoid carcinoma, [5,6,8,9,10,11,12]. ER stress may not induce cell death because the downstream pathways of ER stress vary between cells in cancers depending on the chronicity of ER stress and on the relative expression of key factors [1]. The cell death induced by ER stress can reduce the tumor mass and decrease nutrient and oxygen requirements [1]. In addition, activation of the UPR may increase autophagy, which has a cytoprotective function during stress by liberating amino acids from long-lived proteins and removing damaged organelles [1,13]. PERK mediates upregulation of the autophagy factors LC3 and autophagy-related gene 5 via ATF4 and CCAAT/enhancer-binding protein-homologous protein (CHOP) and promotes phagophore formation [1,13]. The IRE1 arm of the UPR is also important for the survival of hypoxic tumor cells [14]. During hypoxia-induced ER stress, IRE1-driven X-box-binding protein 1 splicing increases tumor cell tolerance to hypoxia, whereas loss of this protein impairs hypoxic tumor growth [14]. Further, generation of reactive oxygen species (ROS) during ER stress, while potentially toxic, may help limit tumor growth to match the nutrient supply by initiating DNA damage checkpoints [1]. Excess toxicity from ROS is limited by ATF4-mediated antioxidant pathways [1].

## 2. Histone Deacetylase (HDAC) Inhibitors Induce ER Stress and Cause Cell Death in Cancer Cells

Since ER stress plays an important role in tumor cell survival, targeting ER homeostasis is considered a potential strategy for the management of cancers [7,15]. Various chemotherapeutic agents induce ER stress in cancer cells [16,17]. In addition, chemotherapy may cause drug resistance in cancer cells, and the underlying mechanisms were found to be related to the induction of ER stress tolerance, GRP78-dependent Akt activation, and suppressed activation of caspase-4 and caspase-7 [9,10,18]. In contrast, suppression of GRP78 using inhibitors or siRNA can enhance the chemotherapy-induced tumor cell apoptosis and drug sensitivity of endothelial cells in tumors [4,5,6,8,18].

In recent years, histone deacetylase (HDAC) has been a target of cancer therapy because it catalyzes the reversible acetylation of histones and nonhistone substrates to control the epigenetic and transcriptomic landscape of normal and tumor cells [19]. Some HDAC inhibitors have been developed for the treatment of cancers, such as apicidin, Gd-metallofullerenol nanomaterial, MS-275, M344, *N*-hydroxy-7-(2-naphthylthio) heptanomide (HNHA), panobinostat, *trans*-3,4,5-trihydroxystilbene (resveratrol), romidepsin, *S*-7-oxo-7-(4-phenylthiazol-2-ylamino)-heptyl) 2-methylpropanethioate (PTACH), sodium butyrate, suberoylanilide hydroxamic acid (SAHA), TMP269, trichostatin A (TSA), valproic acid (VPA), and WJ25591, among others (Table 1) [7,15,17,18,20,21,22,23,24,25,26,27,28]. HDAC inhibitors can suppress cellular proliferation, induce apoptosis, and exert anti-metastatic and anti-angiogenic effects in cancers [29,30,31]. They also induce ER stress, cause hyperacetylation of chaperones including GRP78 and affect their function in protein homeostasis, and induce protein misfolding and proteotoxic stress [7,16,18,19,27]. Several class I HDAC inhibitors including MS-275, apicidin, and romidepsin were shown to potently cause ROS-dependent ER stress-induced apoptosis of nasopharyngeal carcinoma cells [23]. In p53-deficient human colon cells, HDAC inhibitors including sodium butyrate, MS-275, M344, TSA, SAHA, and VPA activated ER stress sensor PERK and eukaryotic translational initiation factor 2α (eIF-2α) phosphorylation, as well as induced the ATF4/ATF3/CHOP pathway [26]. HNHA increases Ca^2+^ release from the ER to the cytoplasm and induces ER-stress-dependent apoptosis in papillary and anaplastic thyroid cancer cells [25]. PTACH and SAHA also enhance ER stress, induce cellular apoptosis, and exert antitumor effects in non-small cell lung cancer (NSCLC) cells [7,17]. SAHA up-regulates ER stress-regulated proteins including ATF4, GRP78, and CCAAT/enhancer-binding protein homologous protein in NSCLC [22]. SAHA treatment rapidly induces sustained eIF2α phosphorylation and enhances cisplatin-induced ER stress-mediated apoptosis in oral squamous cell carcinoma cells; inhibition of ER stress by salubrinal, an inhibitor of eIF2α dephosphorylation, ameliorates this cytotoxicity [28]. In addition, SAHA exerts therapeutic effects on breast cancer cells and shows synergistic therapeutic effects with ionizing radiation (IR) compared with either SAHA or IR treatment alone in MCF-7 and MDA-MB-231 human breast cancer cells, or in 4T1 mouse breast cancer cells [21]. The synergistic effects of combined treatment are thought to occur through autophagy, ER stress, and inhibition of DNA repair proteins [21].

The HDAC inhibitor panobinostat also induces apoptosis and ER stress and inhibits the growth of Caki-1, ACHN, and 769-P renal cancer cells [15]. Panobinostat kills renal cancer cells by inhibiting the degradation of unfolded proteins, causing ubiquitinated proteins to accumulate and inducing ER stress [15]. It also increases the levels of phosphorylated eIF-2α, ATF4, and CHOP and causes GRP78 acetylation, which dissociates GRP78 from PERK, and is associated with the activation of a lethal UPR in human breast cancer cells [27]. In addition, GRP78 knockdown sensitizes MCF-7 breast cancer cells to panobinostat-induced UPR and cell death [27]. Similarly, treatment with TSA causes more apoptosis in MDA-MB-435 breast cancer cells and HCT116 colon cancer cells with GRP78 knockdown by siRNA than in wild-type tumor cells [18]. In multiple myeloma cell lines, the selective class IIa HDAC inhibitor TMP269 enhances cytotoxicity, up-regulates ATF4 and CHOP, and induces apoptosis; however, the enhanced cytotoxicity is abrogated by ATF4 knockdown [24]. WJ25591, a hydroxysuberamide derivative, inhibits HDAC1 and cell proliferation in human PC-3 and DU-145 hormone-refractory prostate cancer cells [20]. In addition, the proteasome inhibitor MG-132 dramatically sensitizes WJ2559-induced apoptosis of prostate cancer cells and ER stress contributes to the synergistic effect [20]. Apicidin can induce histone H3 hyperacetylation and reduction of HDAD2 mRNA expression [22]. It causes apoptotic cell death and activates caspase-3, caspase-9, and caspase-12 [22]. In addition, it increases the expression of ER stress-associated proteins, including CCAAT/CHOP, cleavage of activating transcription factor-6α, and phosphorylation of eIF2α in cancer cells [22]. Inhibition of ER stress by CHOP knockdown or using the ER stress inhibitors salubrinal and 4-phenylbutyric acid reduces apicidin-induced cell death [22]. Apicidin also causes cellular apoptosis by ER stress and mitochondrial dysfunction via phospholipase Cγ1 activation, Ca^2+^ release, and ROS accumulation in Neuro-2a neuroblastoma cells [22]. All of these data suggest HDAC inhibitors induce ER stress to cause cancer cell death.

## 3. Reversion-Inducing Cysteine-Rich Protein with Kazal Motifs (RECK) and Regulation of RECK Expression

The reversion-inducing cysteine-rich protein with Kazal motifs (*RECK*) gene encodes a glycosylphosphatidylinositol-anchored glycoprotein of approximately 110 kDa which contains multiple serine protease inhibitor-like motifs [32,33,34]. RECK is expressed ubiquitously in normal tissues and has various functions in tissue development, morphogenesis, remodeling, tissue architecture, cell migration, cell-cell interaction, chondrogenesis, myogenesis, and angiogenesis [29,33]. It regulates the function of the extracellular matrix and suppresses the activity of matrix metalloproteinases (MMPs), including MMP-2, MMP-9, and membrane type-1, through direct inhibition of its protease activity, regulation of cellular release, and sequestration at the cell surface [34,35]. In addition, the expressions of RECK and MMPs are inversely correlated [36]. Low *RECK* expression is strongly associated with high expression of MMP-2 and MMP-9 in different types of cancers [29,34,37]. *RECK* is considered to be a tumor and metastasis suppressor gene [32,33,34]. RECK expression is reduced in various cancer types including breast, colorectal, lung, pancreatic, prostate, and stomach cancer and cholangiocarcinoma, ameloblastic tumor, middle ear squamous cell cancer, and osteosarcoma [29]. In addition, RECK expression is positively correlated with the survival of cancer patients; down-regulation of RECK often predicts poor prognosis in cancer patients [29]. Restoration of RECK expression in tumor cells suppresses the angiogenesis, invasion, and metastasis of tumors [34,35].

RECK expression is affected by multiple factors. The specificity protein 1 (SP1)-binding site of the *RECK* promoter gene is a common negative target for oncogenic signals [38]. RECK expression is decreased upon cell transformation by human epidermal growth factor receptor 2 (HER-2/neu) and rat sarcoma (RAS) oncoproteins [39,40,41,42]. HER-2/neu induces the binding of SP proteins and HDAC1 to the *RECK* promoter to repress RECK and activates the extracellular signal-regulated kinase signaling pathway [41]. RAS suppresses RECK through inhibition of the SP1 promoter site of the *RECK* gene and via histone deacetylation and promoter methylation mechanisms [39,40]. Further, retinoblastoma binding protein-7, the Ha-RAS (val12)-upregulated gene, forms a complex with HDAC1 and Sp1, which binds to the Sp1 binding site of the *RECK* promoter to suppress RECK expression in 7–4 cells (derived from mouse fibroblast NIH3T3 cells) [43]. Therefore, the SP1 site of the *RECK* promoter is important for the function of RECK.

Histone acetylation/deacetylation plays a key role in the epigenetic regulation of multiple genes [44]. RECK expression is frequently silenced in aggressive tumor cells by HDAC, and suppressed by HER-2/neu and RAS also through a histone deacetylation mechanism [39,40,41,44,45]. The amount or activity of extracellular matrix-degrading enzymes such as MMPs can be modulated by regulating RECK or at the transcriptional and translational levels using HDAC inhibitors [46]. In contrast, RECK expression can be restored by suppressing HDAC with HDAC inhibitors or siRNA [31,39,44,45,46]. Hypoxia-induced down-regulation of RECK is also abolished by knockdown of HDAC1 with siRNA [42]. Further, HDAC inhibitors such as TSA can up-regulate RECK via transcriptional activation in CL-1 human lung cancer cells, as well as rescue hypoxia-suppressed RECK expression in the H-Ras-transformed human breast MCF10A and HT1080 human fibrosarcoma cell lines [31,45]. TSA antagonizes the inhibitory action of Ras on RECK and reverses angiotensin-II-induced RECK suppression by inhibiting Sp1 binding to the RECK promoter [39,44]. Apicidin, which is also a HDAC inhibitor, markedly decreases HDAC4 expression, blocks cell migration and invasion of human ovarian cancer SKOV-3 cells, and suppresses the growth of SKOV-3 xenografts [47]. Apicidin inhibits cell migration through down-regulation of MMP-2 and up-regulation of RECK in HDAC4-blocked SKOV-3 cells [47]. Further, apicidin significantly suppresses the binding of HDAC4 to Sp1 binding elements of the RECK promoter by repressing HDAC4 [47]. Valproic acid induces cytotoxicity and apoptosis and suppresses the invasiveness of T98G glioma cells by up-regulating RECK expression and inhibiting MMP-2 and MMP-9 activity [30]. Gd-metallofullerenol nanomaterial can suppress pancreatic cancer metastasis through down-regulation of metastasis-associated protein 1, HDAC1, hypoxia-inducible factor 1α, and MMP-2/9, and up-regulation of RECK [48]. These data suggest that HADC inhibitors regulate RECK expression and activity via the SP1 binding site of the promoter and affect cancer cell survival.

## 4. HDAC Inhibitors, RECK, and ER Stress

As described above, HDAC inhibitors can induce ER stress, exert antitumor effects, and induce RECK expression in tumor cells; however, the role of RECK in HDAC inhibitor-induced ER stress is unclear. In H460 NSCLC cells, overexpression of microRNA-200c (miR-200c) can suppress cell growth by targeting RECK, followed by activation of the c-jun-N-terminal kinase signaling pathway and ER stress with increased GRP78 and CHOP [49]. Resveratrol, a natural polyphenolic extracted from red wine, is also an HDAC inhibitor and can induce ER stress in miR-200c-transfected H460 NSCLC cells [49]. In addition, resveratrol enhances RECK, GRP78, CHOP, JNK, c-jun, caspase-3, and caspase-9 expression in miR-200c-transfected cells but not in untransfected cells [49]. These findings suggest that miR-200c overexpression can induce ER stress and sensitize H460 cells to resveratrol, which is thought to occur because of increased RECK expression [49]. RECK and GRP78 were shown to colocalize in the cytoplasm and perinuclear area in neuroblastoma cells, indicating that RECK and GRP78 are colocalized in the ER because the glycosylphosphatidylinositol-anchored RECK is transported from the ER to the plasma membrane through the Golgi apparatus [16,34]. Further, RECK overexpression can induce ER stress, as demonstrated by the increased level of phosphorylated PERK and eIF-2α, and exert a cytotoxic effect in neuroblastoma cells [16]. In contrast, GRP78 overexpression inhibits the RECK-induced expression of phosphorylated PERK and eIF-2α in neuroblastoma cells [16]. These findings suggest that RECK binds to and sequesters the GRP78 protein, and transmembrane sensors, such as PERK are released to activate eIF-2α phosphorylation and enhance ER stress in neuroblastoma cells [16]. Collectively, HDAC inhibitors may act on the SP1 binding site of the *RECK* promoter to increase RECK expression. Due to the colocalization and interaction of RECK and GRP78, the increased RECK binds to and sequesters GRP78, eventually activating ER stress.

## 5. Conclusions

**Figure 1:**
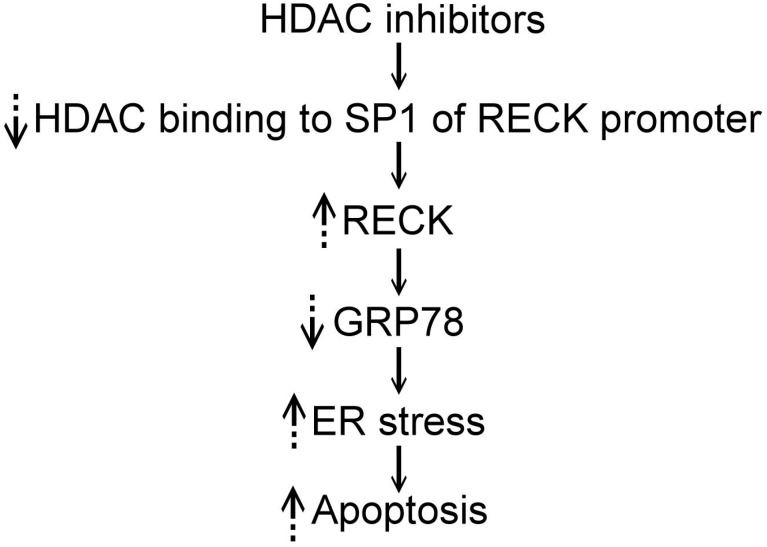
The pathway of the influence of HDAC inhibitors on the ER stress in the tumor cells is speculated as: HDAC inhibitors prevent the binding of HDAC to the SP1 site of the RECK promoter and then increase RECK expression; the increased RECK sequesters GRP78 and eventually activates ER stress and causes cellular apoptosis. ↓ indicates pathway. ↑ indicates increased. ↓ indicates decreased.

In cancers, microenvironmental conditions, like hypoxia, reactive oxygen species, and nutrient deprivation, may lead to the accumulation of unfolded or misfolded proteins and induce ER stress [2,3,4,6,7]. If the ER stress is not relieved, the tumor cells may become apoptotic. Therefore, targeting ER homeostasis is a potential strategy for cancer treatment. HDAC inhibitors can induce ER stress to cause cell death in cancers [7,16,18,27]. In addition, HDAC inhibitors have been found to up-regulate RECK expression by preventing HDAC binding to the SP1 site of the *RECK* promoter [39,40,41,44,45,47]. RECK was found to colocalize with GRP78 to modulate ER stress by binding to and sequestering GRP78. This causes transmembrane sensors, such as PERK, to be released to activate eIF-2α phosphorylation and enhance ER stress [16]. These findings suggest HDAC inhibitors act on the SP1 binding site of the *RECK* promoter to increase RECK expression; the increased RECK sequesters GRP78 and eventually activates ER stress and causes cellular apoptosis (Figure 1) [16,39,44,47]. Collectively, HDAC inhibitors may directly induce ER stress or indirectly induce stress by up-regulating RECK in cancer cells. However, additional studies are necessary to confirm this hypothesis.

## Figures and Tables

**Table 1:** List of histone deacetylase inhibitors.

Histone Deacetylase Inhibitors
Apicidin
Gd-metallofullerenol nanomaterial
MS-275
M344
*N*-hydroxy-7-(2-naphthylthio) heptanomide (HNHA)
Panobinostat
Romidepsin
*S*-7-oxo-7-(4-phenylthiazol-2-ylamino)-heptyl) 2-methylpropanethioate (PTACH)
Sodium butyrate
Suberoylanilide hydroxamic acid (SAHA)
TMP269
*trans*-3,4,5-trihydroxystilbene (resveratrol)
Trichostatin A (TSA)
Valproic acid (VPA)
WJ25591
